# A Functional Drink Containing *Kaempferia parviflora* Extract Increases Cardiorespiratory Fitness and Physical Flexibility in Adult Volunteers

**DOI:** 10.3390/foods12183411

**Published:** 2023-09-13

**Authors:** Jintanaporn Wattanathorn, Terdthai Tong-Un, Wipawee Thukham-Mee, Natthida Weerapreeyakul

**Affiliations:** 1 Research Institute for High Human Performance and Health Promotion, Khon Kaen University, Khon Kaen 40002, Thailand; terdthai@kku.ac.th (T.T.-U.); meewep@gmail.com (W.T.-M.); natthida@kku.ac.th (N.W.); 2 Department of Physiology, Faculty of Medicine, Khon Kaen University, Khon Kaen 40002, Thailand; 3 Faculty of Pharmaceutical Sciences, Khon Kaen University, Khon Kaen 40002, Thailand

**Keywords:** *Kaempferia parviflora*, cardiorespiratory fitness, physical performance

## Abstract

Owing to the reputation of *Kaempferia parviflora* and the crucial role of oxidative stress on the disturbance of physical fitness, the effect of a functional drink containing *K. parviflora* extract (KP) on the physical fitness of healthy adult volunteers was assessed. Healthy male and female volunteers (19–60 years old) were randomly divided into placebo, KP90, and KP180 groups. All the subjects in KP90 and KP180 were directed to consume a functional drink containing *K. parviflora* extract at doses of 90 and 180 mg per serving per 80 mL, respectively. Parameters of physical fitness, including cardiovascular endurance, muscular strength and endurance, flexibility, and body composition, together with changes in lactate, creatinine kinase, and oxidative stress markers were assessed before the intervention, and at 6 and 12 weeks of intervention. The oxidative stress markers, creatine kinase, and lactate were also measured. Subjects who consumed the developed drink had increased VO_2_ max and improved performance in a timed shuttle run test and 5 min distance run, and exhibited decreased oxidative stress and lactate; therefore, *K. parviflora* extract can be successfully used for developing a KP drink to improve cardiorespiratory fitness and physical performance by improving oxidative stress and lactate.

## 1. Introduction

Currently, the increasing life expectancy of the population, the increases in health concerns, and the demand to improve the quality of life, together with growing health care costs play important roles in driving the growing demands for functional food and functional beverages [1], which are foods or drinks that can provide physiological benefits in addition to its naturally occurring benefits. In Thailand, functional drinks have gained much attention. It has been reported that approximately 90% of consumers consume functional drinks on a regular basis [2]. To meet the consumers’ demand, various functional ingredients have been developed, including herbal extracts. Herbal extracts have been preferred as the functional ingredient in the manufacturing of functional drinks due to the belief that herbal extracts can provide a better benefit with less toxicity and toxicity-related disorders. The developed herbal drinks target abundant health objectives, including preventing diseases and improving psychological and physical fitness. It has been reported that functional drinks target physical fitness improvement and have gained much attention due to their high impact on health-related quality of life (HQOL) [3].

Physical fitness is the ability to perform and sustain daily activities and demonstrate the traits and capacities indicating a low risk of premature development of diseases [4]. It has been regarded as one of the human basic requirements. In addition, it serves as a powerful indicator of health among youth [5], and consists of five components, including cardiovascular endurance, muscular strength and endurance, flexibility, and body composition [6,7]. Accumulative evidence has suggested that a low level of health-related physical fitness is associated with premature mortality induced by various systems, such as cardiovascular, respiratory, and metabolic disorders [8,9,10]. 

Recent studies have demonstrated that oxidative stress and inflammation can disturb physical fitness [11,12]. They can induce tissue injury leading to the poor function of tissues, resulting in reductions in cardiorespiratory fitness, range of motion, particularly around the joints, or flexibility, muscle strength, and endurance; thus, the applications of substances possessing antioxidant and anti-inflammatory effects, such as herbal extracts, as the functional ingredients for developing functional drinks have been the main area of focus.

Krachai Dum, or black ginger or *Kaempferia parviflora* Wall. ex Baker, a plant in the Zingiberaceae family, has been used long-term as medicine in Asian countries for treating allergies, fatigue, sexual dysfunction, and ulcers. Experimental data obtained from in vitro, preclinical, and clinical studies have shown that it can decrease oxidative stress [13,14,15]. It also exhibits anti-inflammatory activity [16]. Our previous study revealed that *K. parviflora* extract can improve physical performance in the elderly [15]. In addition, a recent study has demonstrated that some parameters of physical fitness of soccer players also improved after 12 weeks of consumption of *K. parviflora* extract [17]. Owing to the benefits of *K. parviflora* mentioned above, this raised the possibility that *K. parviflora* extract can be utilized as the functional ingredient for developing a functional drink to target the physical fitness improvement in healthy adult volunteers; thus, this study was set up to explore an 8-week consumption effect of a functional drink containing *K. parviflora* extract on the physical fitness of healthy adult volunteers. The possible underlying mechanisms were also investigated. This study will provide benefits as evidence to support the possibility to use *K. parviflora* as the functional ingredient for developing supplements in the food and beverage industry. 

## 2. Materials and Methods

### 2.1. Preparation of Kaempferia parviflora Extract of the Functional Drink Containinng K. parviflora Extract

The *Kaempferia parviflora* extract used in this study was kindly provided by PTT Public Company Limited, Petchabun province, Thailand. In brief, the total rhizomes of *K. parviflora* (var. Khek Noi) at 8 months of growth were harvested during August 2020. After an authentication by a taxonomist of the Ministry of Agriculture and Cooperatives, Thailand, they were prepared as water extract (details are listed under the petty patent registration). The storage conditions were 25 °C in the dark. The extraction was performed within 5 days after harvesting. The extract contained total flavonoids around 3.966%, consisting of 5,7 dimethoxyflavone 2.626%, trimethoxyflavone 0.861%, and pentamethoxyflavone 0.479%, respectively. The flavones in the samples were identified by comparing the retention time and spectral matching with the standards. The flavone standards used in this study were 5,7-dimethoxyflavone, 4′,5,7-trimethoxyflavone, 6,2′,4′-trimethoxyflavone, and 5,7,3′,4′-tetramethoxyflavone. A high-performance liquid chromatography (HPLC) analysis was performed using an LC–2030C3D quaternary pump (Shimadzu, Kyoto, Japan) equipped with a diode array detector (DAD) [16,17]. The sample solutions were injected onto a HiQ sil C18W column (4.6 × 250 mm, 5 µm). The injection volume was 20 µL. A gradient of solvent A (0.5% formic acid in water) and solvent B (acetonitrile) was used at a flow rate of 0.8 mL/min. The column temperature was set at 25 °C. The gradient elution was performed as follows: solvent B 30–40% from 0 to 40 min, followed by solvent B 40–50% from 40 to 60 min; solvent B 50–30% was run from 60 to 65 min and, finally, solvent B 30% was run from 65 to 70 min to monitor the polymethoxyflavone content. Then, *K. parviflora* extract was mixed with maltodextrin, inulin, brown sugar, honey, and heated water (60 °C). After mixing, the mixture was heated at 75 °C for 30 s, poured into a pasteurized container, and CO_2_ was added. The KP90 contained *K. parviflora* extract at concentrations of 90 and 180 mg per serving.

### 2.2. Determination of Total Phenolic Content

The total phenolic content (TPC) was determined by the Folin–Ciocalteu method. Briefly, Folin–Ciocalteu reagent was mixed with the samples in a 96-well plate. After 5 min, Na_2_CO_3_ (solution concentration of 60 g/L) was added, and the mixture was incubated in dark conditions at room temperature for 90 min. The absorbance was measured at 725 nm by a microplate reader. The TPC content was expressed as the gallic acid equivalent (GAE) in mg per gram of the sample [18].

### 2.3. Determination of Total Anthocyanin

The total monomeric anthocyanin was determined by using modifications of the pH differential method in a 96-well plate. In brief, the investigated samples were diluted 10 times with two different buffers (pH 1.0 KCl buffer and pH 4.5 CH_3_CO_2_Na buffer), shaken under dark conditions for 15 min, and centrifuged at room temperature at 2500× *g* 10 min. The absorbance was measured at 510 and 700 nm by a microplate reader [18]. The results were expressed as mg of cyanidin 3-glucoside equivalent per gram of sample.

Total anthocyanin content (mg/g) = A_diff_ × Mw × DF × 1000/ε

where A is (A_510_ − A_700_) _pH 1.0_ − (A_510_ − A_700_) _pH 4.5_, Mw is the molecular weight of cyanidin–3–glucoside (g/mol), DF is the dilution factor (10), and ε is the molar extinction coefficient for 26,900 L mol^−1^ cm^−1^.

### 2.4. Determination of Total Flavonoids Content

The determination of the total flavonoid content (TFC) of the samples was performed using a colorimetric method. The investigated samples in the 96-well plate were mixed with 2% aluminum chloride and incubated at room temperature for 60 min. An absorbance of the reaction mixtures was measured against a blank at 415 nm by a microplate reader. Quercetin was used as the standard [18]. The results were expressed as mg quercetin equivalent (QE)/g of sample.

### 2.5. Study Design

This study was 12 weeks, 3-arm randomized, double-blind, placebo-controlled, parallel group study, and was conducted at the Faculty of Medicine, Khon Kaen University. It was set up to prove the hypothesis whether *K. parviflora* could be used as the functional ingredient for enhancing physical fitness of healthy adults. This study was performed according to the guidelines laid down in the Declaration of Helsinki, and all the procedures involving human subjects were approved by the Institutional Review Board of the Center for Ethics in Human Research, Khon Kaen University, Khon Kaen province, Thailand (HE641265), which corresponds to the Declaration of Helsinki. The protocol was also registered with the Thai Clinical Trials Registry (TCTR 20210528002).

A total of 87 male and female healthy adult volunteers ages 19–60 years old in Amphoe Muaeng, Khon Kaen province were recruited to participate in this study. Advertisements were placed in the local community. The body mass index (BMI) was in the range between 18–25. After screening for eligibility by a semi-structured interview and a physical examination by a physician, 18 subjects were excluded from the study due to ineligibility. The inclusion criteria included healthy men and women at the age mentioned above. Individuals who smoked more than 10 cigarettes per day, had a diagnosis of alcohol addiction or allergies, athletes or people who exercised more than 3 times per week, those who participated in other projects, and people who consumed other supplements were excluded. All the eligible subjects were randomly divided into 3 separate groups, as follows: (1) placebo, (2) KP90, and (3) KP180. The subjects who were assigned to KP90 and KP180 in this study received a developed functional drink that contained *K. parviflora* extract at doses of 90 and 180 mg per serving per day. The placebo also contained all the ingredients as mentioned earlier in the preparation of the functional drink containing *K. parviflora* extract in 2.1, except the extract of *K. parviflora*. Both the placebo and the functional drinks used in this study had the same appearance and flavor. All the subjects in this study provided written consent forms before participating in this study. During the experiments, 4 subjects in the placebo group, 3 subjects from the KP90 group, and 2 subjects from the KP180 group withdrew from the projects due to COVID-19 infection. Finally, a total of 60 male and female subjects participated in the study until the end of 12-week study period. The subjects were required to consume 80 mL of the assigned drink within 5 min for 12 weeks. 

**Figure 1:**
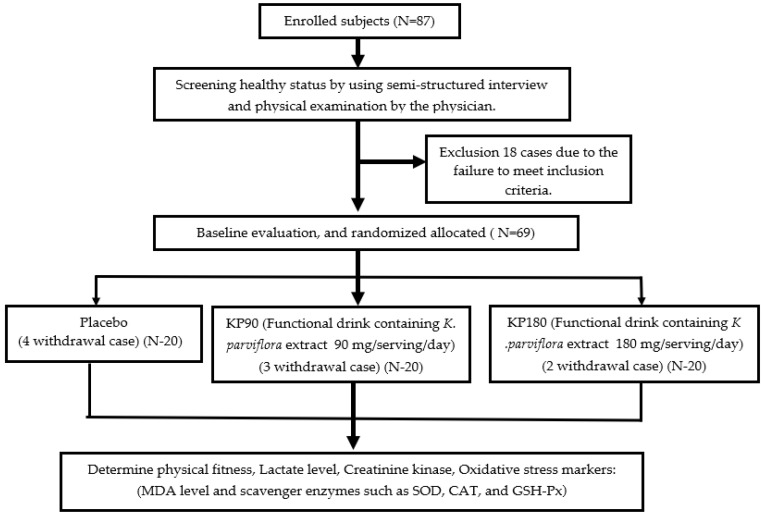
Schematic diagram of the experimental procedures.

All the subjects were assessed for physical fitness, lactate level, creatinine kinase, and oxidative stress status, including the malondialdehyde (MDA) level and the activity of main scavenger enzymes, such as superoxide dismutase (SOD), catalase (CAT), and glutathione peroxidase (GSH-Px) in the serum before the intervention and at 6 and 12 weeks of consumption [19]. A schematic diagram of the experimental procedures is shown in Figure 1. 

### 2.6. Physical Fitness Assessment

#### 2.6.1. Assessment of Maximal Aerobic Capacity (VO_2_ max)

In this study, the maximal aerobic capacity or VO_2_ max was calculated by using the Harvard step test. The Harvard step test is one of the validated tests that is used for predicting VO_2_ max [20]. During the test, all the subjects wore light clothing. After a 5 min rest, they were exposed to the step-test exercise by using a step with a height of 41 cm without shoes. They had to place the right foot on the platform when the signal started, and the left foot was placed on the platform when the second signal was emitted. Then, both legs and the backbone were straightened. The signal “up” was provided at 2 s intervals. Then, the right and left foot were placed on the floor with the third and fourth signal sounds, respectively. All the subjects had to perform this test as long as they could but not exceed 5 min. After the cessation of exercise, the post-exercise heart rate was monitored within 30 s. The VO_2_ max or maximum oxygen uptake was calculated by using the ratio between the maximal heart rate value during exercise (HR_max_) and the heart rate value at rest (HR_rest_) [21] according to the following equation.

VO_2_ max (mL·kg^−1^·min^−1^) = 15 × (HR_max_/HR_rest_) while HR_max_ = 220 − Age.

#### 2.6.2. Assessment of Physical Performance 

In this study, physical performance was assessed by using a battery of tests consisting of a timed shuttle run, sit-ups, push-ups, 5 min distance run, standing long jump tests, and a repeated linear sprint test.

**Multistep 10 m shuttle run test.**

To measure cardiopulmonary endurance, a 10 m shuttle run test, one of the validated methods [22], was applied. This test could also measure the speed of movement, agility, and coordination (motor skills) of the subjects. After a 5 min resting period, the subject ran between 2 markers that had been set up along a 10 m distance with an increment speed triggered by a signal that was played by a standard CD player. During the test, each subject was accompanied by a trained staff member to provide them with an audio signal and to encourage them to go a little faster at the end of each level. The total exercise time was measured and used for analysis [23].

**Standing long jump test.**

In this study, a standing long jump test, a test for assessing the motor coordination of both the upper and lower body segments [24], was applied; however, it is often used for assessing the power of the leg extensor muscles. Each subject jumped with their legs close to each other and a bilateral takeoff was assisted by swinging of the upper body and arms. After the bilateral landing, the distance between the starting and landing points was recorded [24].

**Sit-up test.**

This test was performed to measure the dynamic endurance of the abdominal and hip flexor muscles. During the test, each subject laid on the floor supine with their hands behind their neck while their knees were flexed at an angle of 90°. Then, the subject raised the upper body until the elbows touched the knees, followed by returning to the starting position, where both scapulas touched the floor. The number of completed performances within 60 s was recorded and analyzed [25].

**Push-up test.**

This test was performed to assess the overall upper body performance, particularly the anterior shoulder strength and stability, and the ability to produce high forces during the pushing activity. The subjects pressed themselves with their arms fully extended and lowered themselves back down until their chests were three inches from the floor. Then, they repeated the process as many times as they could within one minute. The number of completed processes within 1 min was measured and used for data analysis [26].

**Five-minute distance run test.**

This test was used for assessing aerobic endurance. Markers were placed at set intervals around a track for assisting the measurement of the completed distance. Each subject had to run for 5 min, and the total distance was recorded [27].

**Repeated linear sprint test.**

Each subject performed a 6 × 40 m repeated sprint test [28]. The interval between each test was set up at 20 s. The best running speed, average speed, and percentage of decrement speed were recorded. 

### 2.7. Biochemical Assessments

The lactate level was measured using a colorimetric assay kit (Cell Biolabs’ Lactate Assay Kit), whereas the creatinine kinase activity was determined using a Creatine Kinase Activity Assay Kit (ab155901) at Srinagarind Hospital, Faculty of Medicine, Khon Kaen University, Khon Kaen, Thailand. Oxidative stress markers, including the malondialdehyde (MDA) level, and the activities of superoxide dismutase (SOD), catalase (CAT), and glutathione peroxidase (GSH-Px) were measured as previously mentioned [19]. In brief, the MDA was monitored using a thiobarbituric acid reactive substances assay (TBARS) method, whereas the SOD was measured by the reduction of nitroblue tetrazolium with a superoxide generator induced by a xanthine–xanthine oxidase reaction, and the CAT was assessed using the decomposition of H_2_O_2_ as an indicator. GSH-Px was also monitored by measuring the oxidation of NADH to NADP by enzyme glutathione reductase during the oxidation of oxidized glutathione (GSSG), which is formed by the catalytic action of organic peroxide by GSH-Px [29].

### 2.8. Statistical Analysis

All the data were presented as mean ± standard error of mean (SEM). The normality of the data was assessed using Kolmogorov–Smirnov tests. A one-way analysis of variance (ANOVA) was used to determine the difference between the groups, followed by Tukey’s post hoc test. The statistical difference was regarded when the *p*-value was less than 0.05.

## 3. Results

### 3.1. Contents of the Polymethoxyflavone, Polyphenol, and Flavonoids

The total phenolic compounds, anthocyanins, and flavonoids of KP90 were 0.212 ± 0.005 μg GAE/mg sample, 0.013 ± 0.001 μg C3G/mg sample, and 0.184 ± 0.004 μg Quercetin/mg sample, whereas those in KP180 were 0.364 ± 0.013 μg GAE/mg sample, 0.025 ± 0.001 μg C3G/mg sample, and 0.216 ± 0.005 μg Quercetin/mg sample, respectively.

**Figure 2:**
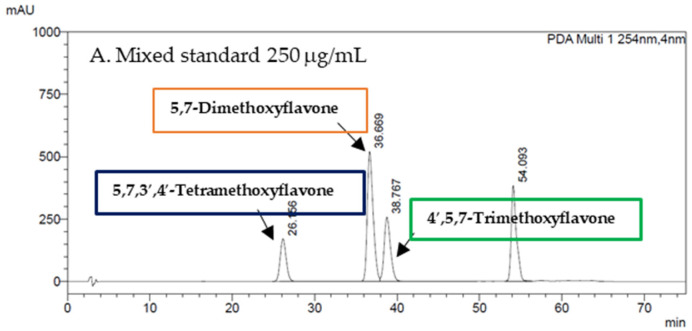

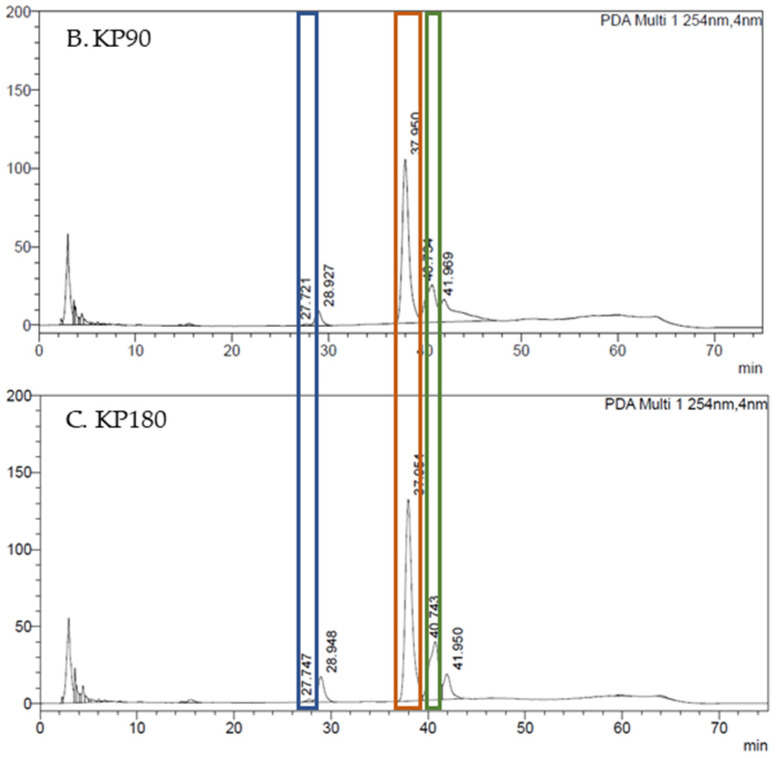
Fingerprint chromatogram of 5,7-dimethoxyflavone, 4′,5,7-trimethoxyflavone, and 5,7,3′,4′ -tetramethoxyflavone and the contents of the mentioned substances in KP90 and KP180.

Based on a previous study, polymethoxyflavone has been claimed as the possible active ingredient of *K. parviflora* [18]. We also measured the concentration of the methoxyflavone content in the KP90 and KP180 using an HPLC method for quality control. Our data reveal that the developed KP90 contained 5,7-dimethoxyflavone, 4′,5,7-trimethoxyflavone, and 5,7,3′,4′-tetramethoxyflavone at concentrations of 0.054 ± 0.0043, 0.024 ± 0.0016, and 0.0013 ± 0.000012 μg/mL, respectively, whereas the concentrations of these substances in KP180 were 0.066 ± 0.0009, 0.034 ± 0.0023, and 0.0023 ± 0.00009 μg/mL, respectively, as shown in Figure 2.

### 3.2. General Characteristic of Subjects

Table 1 reveals that before the intervention, no significant differences in age, gender, vital signs (body temperature, heart rate, respiratory rate, systolic and diastolic blood pressure), body weight, height, and body mass index (BMI) among the groups were observed. After the consumption of the assigned product for 6 and 12 weeks, all these parameters still failed to show significant differences among the groups, as shown in Table 2 and Table 3.

We also assessed the effect of the functional drink containing *K. parviflora* extract on the body composition, one of the components of physical fitness. It has been revealed that no significant differences in body composition were observed before the intervention and throughout the study period, as shown in Table 4, Table 5 and Table 6.

### 3.3. Effect of the Functional Drink Containing K. parviflora Extract on Physical Fitness

**Figure 3:**
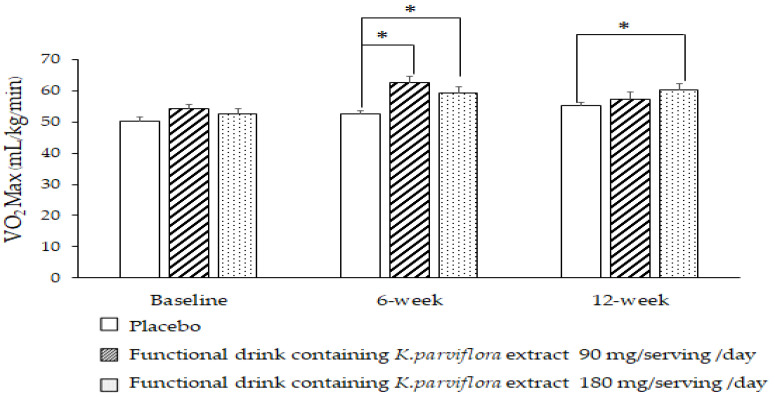
Effect of the functional drink containing *K. parviflora* extract at doses of 90 and 180 mg/serving/day on the cardiorespiratory endurance assessed using VO_2_ max assessment via Harvard step test. Data are presented as mean ± SEM. (*n* = 20/group). * *p*-value < 0.05, compared to placebo group.

Figure 3 shows the effect of the functional drink containing *K. parviflora* extract on cardiovascular endurance, which was assessed using VO_2_ max as an indicator. Our data show that no significant difference in VO_2_ max was observed at the baseline level. After 6 weeks of consumption, the subjects who consumed the functional drink at doses of 90 and 180 mg per serving per day demonstrated significantly increased VO_2_ max values (*p*-value < 0.05, all compared to the placebo group). When the consumption was prolonged to 12 weeks, a significant increase in this parameter was observed only in subjects who consumed the high dose of the functional drink containing *K. parviflora* (*p*-value < 0.05, compared to the placebo group).

To assess the effect of the functional drink on physical performance, the performance of all the subjects in a timed shuttle run, standing long jump, sit-up, push-up, and 5 min distance run tests were determined before the intervention, after 6 weeks of consumption, and after 12 weeks of consumption. The results are shown in Table 7. It was revealed that none of the subjects showed significant differences in all the measured parameters. After 12 weeks of consumption, the subjects who consumed the high dose of the developed functional drink displayed improved performance in both the timed shuttle run test and the 5 min distance test (*p*-value < 0.05, all compared to the placebo group).

### 3.4. Effect of the Functional Drink Containing K. parviflora Extract on Biochemical Parameters

**Figure 4:**
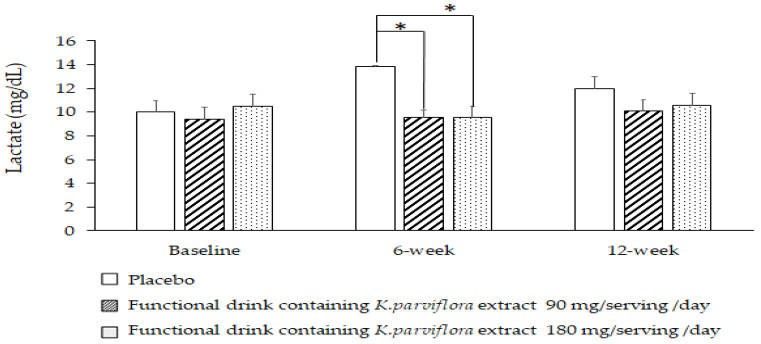
Effect of the functional drink containing *K. parviflora* extract at doses of 90 and 180 mg/serving/day on serum lactate level. Data are presented as mean ± SEM. (*n* = 20/group). * *p*-value < 0.05, compared to placebo group.

The results reveal that there was no significant difference in the serum lactate level among the groups before the experiment. At the 6-week consumption period, subjects who consumed the developed functional drink at both doses used in this study showed a significant reduction in the serum lactate level (*p*-value < 0.05, all compared to placebo); however, no significant changes of this parameter were observed after the 12-week consumption period, as shown in Figure 4.

**Figure 5:**
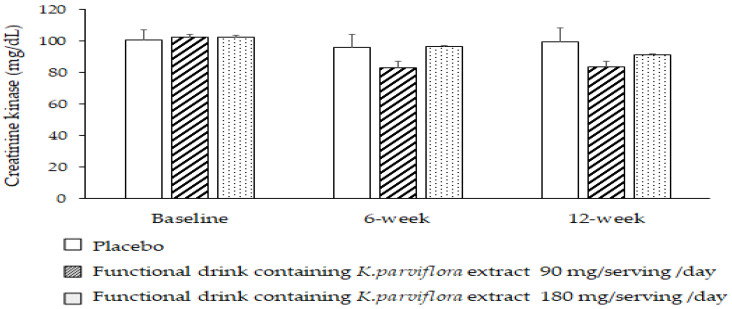
Effect of the functional drink containing *K. parviflora* extract at doses of 90 and 180 mg/serving/day on serum creatinine kinase activity. Data are presented as mean ± SEM. (*n* = 20/group).

The effect of the developed functional drink on creatinine kinase was also explored, and the results are shown in Figure 5. No significant changes of this parameter were observed at the baseline level or after the 6- and 12-week consumption periods.

The serum oxidative stress markers, including the malondialdehyde (MDA) level, and the activities of the main scavenger enzymes, such as SOD, CAT, and GSH-Px, of all the subjects who consumed the assigned substances were also investigated, and the data are shown in Table 8. It was found that none of the parameters mentioned above showed significant changes among the groups at the baseline level. At 6 weeks of consumption period, the subjects who consumed both doses of the functional drink significantly increased the activities of SOD and CAT (*p*-value < 0.05, all compared to the placebo group). The significant reduction in the MDA level after 6 weeks of consumption was observed only in the subjects who consumed the high dose of the developed functional drink (*p*-value < 0.05, compared to the placebo group). When the consumption was prolonged to 8 weeks, it was found that the subjects who consumed the functional drink at both doses used in this study had a decreased MDA level (*p*-value < 0.01, all compared to the placebo group). The significantly increased activity of SOD after 8 weeks of consumption was observed only in the subjects who consumed the low dose of the developed functional drink (*p*-value < 0.05, compared to the placebo group).

## 4. Discussion

The current study clearly reveals that the subjects who consumed the functional drink containing *K. parviflora* extract showed improvement in VO_2_ max and performance in the timed shuttle run and 5 min distance run tests. The reduction in MDA and lactate levels together with the elevation of SOD and catalase were also observed.

VO_2_ max is regarded not only as a cardiorespiratory fitness index, but also as a major determinant of endurance performance [30]. Our data clearly demonstrate that the functional drinks containing an extract of *K. parviflora* at doses of 90 and 180 mg per day produce a significant increase in this parameter. The improvement of VO_2_ max observed in this study also corresponds with the previous work that demonstrates the improvement of this parameter after the consumption of a *K. parviflora* extract capsule at a dose of 180 mg per day after 8 weeks of consumption [31]. The improved performances in both the timed shuttle run and 5 min distance run were also observed in subjects who consumed the high dose of the developed functional drink. It has been reported that physical performance, particularly the timed shuttle run, can measure the speed of movement, agility, coordination (motor skills), and cardiorespiratory fitness, which are associated with oxidative stress status [32,33,34]. Furthermore, both the timed shuttle run test and 5 min distance run test can also measure the aerobic capacity of the body [35,36]; therefore, these results confirm that the antioxidative properties of this substance can enhance the capacity of the body to neutralize oxidative stress, which can disturb mitochondria function [37], giving rise to higher ATP production, higher VO_2_ max, and better exercise performance in activities such as the timed shuttle run and 5 min distance run [38,39]. The mentioned changes also correspond with our results, which show a significant increase in SOD and CAT but a decrease in the MDA level; however, at 8 weeks of the consumption period, the elevation in SOD fails to show the closed association with the MDA level, and no significant changes in the CAT or GPS-Px enzymes were observed. The possible explanation may derive partly from the changes of other scavenger enzymes and non-enzymatic antioxidants. In addition, the reduction in oxidative stress formation can possibly contribute a role; however, understanding of the detailed mechanisms still requires further exploration.

During exercise, lactate production from glycolysis is enhanced until it is in excess and clearance is required. The accumulated lactate, in turn, disturbs muscle performance [40,41]. The present study has demonstrated that the serum lactate level of the subjects who consumed a functional drink containing *K. parviflora* extract at both doses was reduced after 6 weeks of consumption. When consumption is extended to 12 weeks, this change still shows a reduction trend, but no significant change is present. These data suggest that the functional drink may possibly increase lactate clearance.

Our study clearly demonstrates that *K. parviflora* extract can improve physical fitness and performance. These findings correspond with the previous findings, which show the improvement in some physical work capacity [19,31]. Owing to the effect of dimethoxyflavone on the increase in muscle performance via an increase in mitochondria function and antioxidant activity [42], we do suggest that the possible active ingredient may occur partly via the dimethoxyflavone present in *K. parviflora* extract; however, the crucial role of 4,5,7-trimethoxyflavone on vasodilation [43], which, in turn, plays an important role in renal clearance [43], still cannot be omitted. *K. parviflora* appears to be a good candidate for physical fitness enhancement with safety in doses up to 1.35 g per day [44]. Our findings suggest that *K. parviflora* can provide a benefit as the potential functional ingredient for developing functional beverages and functional food, which, in turn, provide great impacts on commercial and industrial aspects. Moreover, it can also provide health benefits, which, in turn, decrease medical costs of the country and increase the productivity of human resources of the country.

## 5. Conclusions

This study is the first study to demonstrate that a functional drink containing *K. parviflora* extract can improve cardiorespiratory fitness and some physical performance. The possible underlying mechanism may be partly associated with the reduction of oxidative stress and serum lactate. According to these results, it can be suggested that *K. parviflora* extract may possibly be used as an herbal-derived functional ingredient for physical fitness promotion. It may be further applied to protect against oxidative stress-related disorders. There are, however, some limitations of this study, as follows: (1) the number of subjects was limited and (2) the assessment of VO_2_ max was performed indirectly via calculation due to the COVID-19 pandemic; therefore, confirmation by a clinical trial study with more subjects and direct measurements will increase the strength of the supported evidence for the health benefits of *K. parviflora* extract on physical fitness.

## Figures and Tables

**Table 1:** General characteristics of volunteers, including age, gender, body temperature, heart rate, systolic and diastolic blood pressure, body weight, body height, and body mass index (BMI) before the intervention. Data are presented as mean ± SEM. (*n* = 20/group).

Parameters	Baseline		
	Placebo	Functional Drink Containing *K. parviflora* Extract at a Dose of 90 mg/Serving/Day	Functional Drink Containing *K. parviflora* Extract at a Dose of 180 mg/Serving/Day
Age (year)	30.58 ± 1.55	33.29 ± 1.87(*p* = 0.300)	30.44 ± 1.45 (*p* = 0.882)
Gender (Male/Female)	7/13	7/13	7/13
Body Temperature (°C)	36.55 ± 0.03	36.51 ± 0.04 (*p* = 0.512)	36.38 ± 0.08 (*p* = 0.126)
Heart Rate (beats/min)	81.05 ± 3.29	75.00 ± 2.25 (*p* = 0.104)	73.94 ± 2.02 (*p* = 0.054)
Respiratory Rate (breaths/min)	16.17 ± 0.21	16.47 ± 0.19 (*p* = 0.346)	16.44 ± 0.20 (*p* = 0.381)
Systolic BP (mmHg)	109.23 ± 3.44	112.58 ± 2.18 (*p* = 0.416)	113.50 ± 2.80 (*p* = 0.295)
Diastolic BP (mmHg)	72.52 ± 2.10	71.17 ± 2.71 (*p* = 0.680)	69.27 ± 1.99 (*p* = 0.316)
Body Weight (kg)	62.95 ± 1.85	61.42 ± 3.87 (*p* = 0.185)	63.44 ± 3.20 (*p* = 0.908)
Body Height (cm)	166.06 ± 1.83	162.42 ± 2.20 (*p* = 0.212)	160.92 ± 2.15 (*p* = 0.086)
Body Mass Index (kg/m^2^)	23.07 ± 0.51	22.68 ± 0.98 (*p* = 0.726)	23.54 ± 0.74 (*p* = 0.669)

**Table 2:** General characteristics of volunteers, including age, gender, body temperature, heart rate, systolic and diastolic blood pressure, body weight, body height, and body mass index (BMI) at 6 weeks of intervention. Data are presented as mean ± SEM. (*n* = 20/group).

Parameters	6 Weeks		
	Placebo	Functional Drink Containing *K. parviflora* Extract at a Dose of 90 mg/Serving/Day	Functional Drink Containing *K. parviflora* Extract at a Dose of 180 mg/Serving/Day
Age (year)	30.58 ± 1.55	33.29 ± 1.87 (*p* = 0.300)	30.44 ± 1.45 (*p* = 0.882)
Gender (Male/Female)	7/12	7/13	7/13
Body Temperature (°C)	36.09 ± 0.08	36.09 ± 0.12 (*p* = 0.439)	36.18 ± 0.03 (*p* = 0.715)
Heart Rate (beats/min)	75.33 ± 2.66	76.00 ± 2.72 (*p* = 0.853)	72.30 ± 2.22 (*p* = 0.412)
Respiratory Rate (breaths/min)	16.46 ± 0.13	16.85 ± 0.14 (*p* = 0.061)	16.46 ± 0.18 (*p* = 0.811)
Systolic BP (mmHg)	108.53 ± 3.14	106.28 ± 3.11 (*p* = 0.646)	111.53 ± 3.60 (*p* = 0.661)
Diastolic BP (mmHg)	70.06 ± 2.74	68.00 ± 2.71 (*p* = 0.602)	70.76 ± 3.03 (*p* = 0.862)
Body Weight (kg)	64.19 ± 2.26	61.57 ± 4.39 (*p* = 0.559)	62.59 ± 2.48 (*p* = 0.725)
Body Height (cm)	166.06 ± 1.83	162.42 ± 2.20 (*p* = 0.212)	160.92 ± 2.15 (*p* = 0.086)
Body Mass Index (kg/m^2^)	23.24 ± 0.65	23.01 ± 1.10 (*p* = 0.842)	24.10 ± 0.67 (*p* = 0.474)

**Table 3:** General characteristics of volunteers, including age, gender, body temperature, heart rate, systolic and diastolic blood pressure, body weight, body height, and body mass index (BMI) at 12 weeks of intervention. Data are presented as mean ± SEM. (*n* = 20/group).

Parameters	12 Weeks		
	Placebo	Functional Drink Containing *K. parviflora* Extract at a Dose of 90 mg/Serving/Day	Functional Drink Containing *K. parviflora* Extract at a Dose of 180 mg/Serving/Day
Age (year)	30.33 ± 1.75	33.50 ± 2.20 (*p* = 0.284)	30.69 ± 1.90 (*p* = 0.926)
Gender (Male/Female)	7/12	7/13	7/13
Body Temperature (°C)	36.16 ± 0.03	36.27 ± 0.07 (*p* = 0.098)	36.15 ± 0.03 (*p* = 0.790)
Heart Rate (beats/min)	77.06 ± 2.59	76.71 ± 2.42 (*p* = 0.917)	75.30 ± 2.13 (*p* = 0.611)
Respiratory Rate (breaths/min)	16.46 ± 0.16	16.64 ± 0.19 (*p* = 0.523)	16.53 ± 0.24 (*p* = 0.581)
Systolic BP (mmHg)	108.80 ± 3.32	111.85 ± 3.51 (*p* = 0.496)	111.92 ± 2.56 (*p* = 0.496)
Diastolic BP (mmHg)	71.73 ± 2.23	71.57 ± 1.98 (*p* = 0.963)	67.38 ± 3.19 (*p* = 0.223)
Body Weight (kg)	64.56 ± 2.14	61.35 ± 4.41 (*p* = 0.471)	63.11 ± 2.48 (*p* = 0.749)
Body Height (cm)	166.06 ± 1.83	162.42 ± 2.20 (*p* = 0.215)	161.69 ± 2.20 (*p* = 0.145)
Body Mass Index (kg/m^2^)	23.37 ± 0.59	22.90 ± 1.06 (*p* = 0.661)	24.04 ± 0.51 (*p* = 0.547)

**Table 4:** Body composition of volunteers before the intervention. Data are presented as mean ± SEM. (*n* = 20/group).

Parameters	Baseline		
	Placebo	Functional Drink Containing *K. parviflora* Extract at a Dose of 90 mg/Serving/Day	Functional Drink Containing *K. parviflora* Extract at a Dose of 180 mg/Serving/Day
Water (%)	52.91 ± 1.18	54.18 ± 1.01 (*p* = 0.412)	52.71 ± 1.01 (*p* = 0.895)
Visceral Fat (%)	5.76 ± 0.55	6.11 ± 1.04 (*p* = 0.652)	6.50 ± 0.78 (*p* = 0.559)
Total Fat (%)	27.70 ± 1.62	25.98 ± 1.38 (*p* = 0.409)	28.12 ± 1.33 (*p* = 0.839)
Muscle Mass (%)	43.40 ± 1.82	43.01 ± 2.82 (*p* = 0.361)	43.00 ± 2.35 (*p* = 0.644)
Muscle Mass Fat (%)	4.29 ± 0.31	4.41 ± 0.25 (*p* = 0.650)	3.66 ± 0.32 (*p* = 0.121)
Bone Mass (%)	2.57 ± 0.08	2.45 ± 0.13 (*p* = 0.453)	2.52 ± 0.11 (*p* = 0.738)

**Table 5:** Body composition of volunteers at 6 weeks of the intervention. Data are presented as mean ± SEM. (*n* = 20/group).

Parameters	6 Weeks		
	Placebo	Functional Drink Containing *K. parviflora* Extract at a Dose of 90 mg/Serving/Day	Functional Drink Containing *K. parviflora* Extract at a Dose of 180 mg/Serving/Day
Water (%)	52.96 ± 1.26	53.45 ± 0.86 (*p* = 0.750)	51.19 ± 1.09 (*p* = 0.263)
Visceral Fat (%)	6.06 ± 0.68	6.28 ± 1.19 (*p* = 0.628)	6.07 ± 0.64 (*p* = 0.907)
Total Fat (%)	27.66 ± 1.72	27.00 ± 1.17 (*p* = 0.751)	30.03 ± 1.49 (*p* = 0.271)
Muscle Mass (%)	43.79 ± 1.88	42.40 ± 3.04 (*p* = 0.222)	66.86 ± 25.32 (*p* = 0.695)
Muscle Mass Fat (%)	4.33 ± 0.28	3.85 ± 0.29 (*p* = 0.162)	4.07 ± 0.39 (*p* = 0.845)
Bone Mass (%)	2.62 ± 0.08	2.43 ± 0.15 (*p* = 0.245)	2.48 ± 0.10 (*p* = 0.395)

**Table 6:** Body composition of volunteers at 12 weeks of the intervention. Data are presented as mean ± SEM. (*n* = 20/group).

Parameters	12 Weeks		
	Placebo	Functional Drink Containing *K. parviflora* Extract at a Dose of 90 mg/Serving/Day	Functional Drink Containing *K. parviflora* Extract at a Dose of 180 mg/Serving/Day
Water (%)	53.19 ± 1.30	53.87 ± 0.90 (*p* = 0.666)	50.96 ± 1.11 (*p* = 0.172)
Visceral Fat (%)	6.00 ± 0.65	6.42 ± 1.19 (*p* = 0.878)	6.23 ± 0.61 (*p* = 0.691)
Total Fat (%)	27.34 ± 1.78	26.37 ± 1.22 (*p* = 0.657)	30.38 ± 1.52 (*p* = 0.173)
Muscle Mass (%)	44.20 ± 1.83	42.63 ± 3.13 (*p* = 0.256)	41.41 ± 1.88 (*p* = 0.240)
Muscle Mass Fat (%)	4.66 ± 0.23	4.64 ± 0.34 (*p* = 0.454)	3.84 ± 0.42 (*p* = 0.099)
Bone Mass (%)	2.68 ± 0.08	2.45 ± 0.15 (*p* = 0.179)	2.50 ± 0.10 (*p* = 0.321)

**Table 7:** Effect of the functional drink containing *K. parviflora* extract at doses of 90 and 180 mg/serving/day on various tests of physical performance assessment. Data are presented as mean ± SEM. (*n* = 20/group). * *p*-value < 0.05, compared to placebo group.

Times	Parameters	Placebo	Functional Drink Containing *K. parviflora* Extract at a Dose of 90 mg/Serving/Day	Functional Drink Containing *K. parviflora* Extract at a Dose of 180 mg/Serving/Day
Baseline	Timed shuttle run (s)	7.33 ± 0.33	7.94 ± 0.43 (*p* = 0.568)	7.81 ± 0.28 (*p* = 0.699)
	Standing long jump (m)	1.46 ± 0.10	1.48 ± 0.09 (*p* = 0.743)	1.53 ± 0.09 (*p* = 0.620)
	Sit-up (times)	13.23 ± 0.96	12.17 ± 0.91 (*p* = 0.306)	12.11 ± 0.70 (*p* = 0.514)
	Push-up (times)	17.58 ± 1.39	16.29 ± 1.26 (*p* = 0.545)	14.77 ± 1.34 (*p* = 0.119)
	5 min distance run (m)	515.00± 16.81	494.28 ± 22.03 (*p* = 0.395)	524.17 ± 10.98 (*p* = 0.737)
	Repeated linear sprint test (s)	14.91 ± 0.71	13.78 ± 0.70 (*p* = 0.235)	13.85 ± 0.73 (*p* = 0.306)
6 weeks	Timed shuttle run (s)	7.08 ± 0.33	7.76 ± 0.48 (*p* = 0.756)	7.19 ± 0.41 (*p* = 0.816)
	Standing long jump (m)	1.36 ± 0.09	1.31 ± 0.08 (*p* = 0.512)	1.27 ± 0.09 (*p* = 0.279)
	Sit-up (times)	13.08 ± 1.04	13.08 ± 0.56 (*p* = 1.000)	14.09 ± 0.88 (*p* = 0.413)
	Push-up (times)	16.64 ± 1.48	16.15 ± 1.18 (*p* = 0.770)	15.83 ± 1.41 (*p* = 0.816)
	5 min distance run (m)	490.00 ± 19.90	498.33 ± 21.45 (*p* = 0.688)	516.36 ± 21.37 (*p* = 0.803)
	Repeated linear sprint test (s)	16.76 ± 0.94	16.27 ± 1.06 (*p* = 0.707)	15.92 ± 0.72 (*p* = 0.532)
12 weeks	Timed shuttle run (s)	7.81 ± 0.41	7.19 ± 0.25 (*p* = 0.524)	6.79± 0.22 * (*p* = 0.033)
	Standing long jump (m)	1.51 ± 0.11	1.40 ± 0.08 (*p* = 0.711)	1.38 ± 0.08 (*p* = 0.645)
	Sit-up (times)	13.60 ± 1.12	14.21 ± 1.44 (*p* = 0.895)	14.07 ± 1.02 (*p* = 0.50)
	Push-up (times)	17.40 ± 2.07	18.14 ± 1.38 (*p* = 0.512)	15.07 ± 1.37 (*p* = 0.392)
	5 min distance run (m)	504.00 ± 18.56	502.50 ± 29.11 (*p* = 0.419)	529.09 ± 19.66 * (*p* = 0.049)
	Repeated linear sprint test (s)	17.83 ± 1.02	16.83 ± 0.91 (*p* = 0.861)	15.92 ± 0.56 (*p* = 0.259)

**Table 8:** Effect of the functional drink containing *K. parviflora* extract at doses of 90 and 180 mg/serving/day on oxidative stress status, assessed using serum malondialdehyde (MDA) level and the activities of superoxide dismutase (SOD), catalase (CAT), and glutathione peroxidase (GPx). Data are presented as mean ± SEM. (*n* = 20/group). * *p*-value < 0.05; compared to placebo group, ** *p*-value < 0.01; compared to placebo group.

Time	Parameters	Placebo	Functional Drink Containing *K. parviflora* Extract at a Dose of 90 mg/Serving/Day	Functional Drink Containing *K. parviflora* Extract at a Dose of 180 mg/Serving/Day
Baseline	MDA (ng/mg·protein)	1.57 ± 0.28	1.18 ± 0.16 (*p* = 0.304)	1.56 ± 0.34 (*p* = 0.978)
	SOD (U/mg·protein)	10.49 ± 1.86	11.96 ± 2.39 (*p* = 0.605)	10.56 ± 1.67 (*p* = 0.981)
	Catalase (U/mg·protein)	9.27 ± 1.61	10.10 ± 1.38 (*p* = 0.668)	9.18 ± 0.95 (*p* = 0.961)
	GPx. (U/mg·protein)	0.34 ± 0.07	0.31 ± 0.04 (*p* = 0.708)	0.37 ± 0.05 (*p* = 0.673)
6 weeks	MDA (ng/mg·protein)	1.80 ± 0.21	1.78 ± 0.14 (*p* = 0.909)	1.15 ± 0.11 * (*p* = 0.032)
	SOD (U/mg·protein)	14.20± 3.39	24.66 ± 3.98 * (*p* = 0.032)	26.40 ± 3.08 * (*p* = 0.018)
	Catalase (U/mg·protein)	7.32 ± 0.42	9.68 ± 0.78 * (*p* = 0.042)	10.12 ± 1.04 * (*p* = 0.019)
	GPx. (U/mg·protein)	0.53 ± 0.10	0.61 ± 0.07 (*p* = 0.547)	0.54 ± 0.07 (*p* = 0.936)
12 weeks	MDA (ng/mg·protein)	2.46 ± 0.29	1.55 ± 0.18 ** (*p* = 0.009)	1.55 ± 0.17 * (*p* = 0.016)
	SOD (U/mg·protein)	18.33 ± 2.09	27.70 ± 2.92 * (*p* = 0.031)	23.19 ± 3.80 (*p* = 0.249)
	Catalase (U/mg·protein)	12.73 ± 1.30	14.03 ± 1.48 (*p* = 0.487)	13.69 ± 1.20 (*p* = 0.616)
	GSH-Px. (U/mg·protein)	0.80 ± 0.08	1.07 ± 0.09 (*p* = 0.302)	0.98 ± 0.02 (*p* = 0.503)

## Data Availability

Data is contained within the article.
